# Autophagy regulates odontoblast differentiation by suppressing NF-*κ*B activation in an inflammatory environment

**DOI:** 10.1038/cddis.2015.397

**Published:** 2016-03-03

**Authors:** F Pei, H S Wang, Z Chen, L Zhang

**Affiliations:** 1 The State Key Laboratory Breeding Base of Basic Science of Stomatology (Hubei-MOST) and Key Laboratory for Oral Biomedicine of Ministry of Education (KLOBM), School and Hospital of Stomatology, Wuhan University, Wuhan, China

## Abstract

Odontoblasts are derived from dental papilla mesenchymal cells and have an important role in defense against bacterial infection, whereas autophagy can recycle long-lived proteins and damaged organelles to sustain cellular homeostasis. Thus, this study explores the role of autophagy in odontoblast differentiation with lipopolysaccharide (LPS) stimulation *in vitro* and the colocalization of p-NF-*κ*B and LC3 in caries teeth. The odontoblasts differentiation was enhanced through LPS stimulation, and this outcome was reflected in the increased number of mineralized nodules and alkaline phosphatase (ALP) activity. The expression levels of the autophagy markers LC3, Atg5, Beclin1 and TFE3 increased time dependently, as well along with the amount of autophagosomes and autophagy fluxes. This result suggests that autophagy was enhanced in odontoblasts cultured with mineralized-induced media containing LPS. To confirm the role of autophagy in differentiated odontoblasts with LPS stimulation, chloroquine (CQ) or rapamycin were used to either block or enhance autophagy. The number of mineralized nodules decreased when autophagy was inhibited, but this number increased with rapamycin treatment. Phosphorylated nuclear factor-*κ*B (NF-*κ*B) expression was negatively related to autophagy and could inhibit odontoblast differentiation. Furthermore, p-NF-*κ*B and LC3 colocalization could be detected in cells stimulated with LPS. The nucleus translocation of p-NF-*κ*B in odontoblasts was enhanced when autophagy was inhibited by Atg5 small interfering RNA. In addition, the colocalization of p-NF-*κ*B and LC3 in odontoblasts and sub-odontoblastic layers was observed in caries teeth with reactionary dentin. Therefore, our findings provide a novel insight into the role of autophagy in regulating odontoblast differentiation by suppressing NF-*κ*B activation in inflammatory environments.

Tooth organogenesis is a complex process involving reciprocal interactions between epithelium and mesenchymal cells.^1^ Dentin is the main hard tissue of the tooth that is formed by odontoblasts, which stem from neural crest-derived odontogenic mesenchymal cells.^1, 2^ The dental papilla gives rise to odontoblasts,^2, 3^ and dentinogenic markers dentin sialoprotein (DSP), dentin matrix protein-1 (DMP1) and osterix (OSX) are increased during odontoblast differentiation.^3, 4^ Odontoblasts are responsible for the production of physiological primary and secondary dentin.^5, 6^ Nonetheless, these cells, along with dental pulp tissues, are frequently infected with bacteria from dental caries.^7^ In the event of carious invasion, odontoblasts induce immune inflammatory responses and reactionary dentin to defend against invading pathogens.^8^ When odontoblasts are damaged, the progenitors in the pulp can migrate and differentiate into odontoblast-like cells and form reparative dentin.^8, 9^ Therefore, the behavior of odontoblasts must be explored, and the knowledge obtained may be applied to dental pulp regeneration.

Autophagy is a cellular process that recycles damaged organelles and long-lived proteins to maintain cell energy homeostasis.^10^ This process is initiated by the formation of double membrane vesicles. Subsequently, the lipid-based membrane is elongated, and the cargo is encapsulated to generate mature autophagosomes.^10^ Then, autophagosomes fuse with lysosomes and degrade cellular components to maintain cytoplasmic homeostasis. mTOR as the master regulator negatively regulates autophagy.^11^ Autophagy is also regulated by a series of proteins known as core autophagic machinery, which consists of autophagy-related gene (ATG) products.^12, 13^ Beclin1 is part of the class III PtdIns 3-kinase complex involved in autophagosome formation. Two ubiquitin-like conjugation systems, namely, Atg5–Atg12 and LC3 (microtubule-associated protein-1 light chain-3), are necessary for the elongation and maturation of autophagosomes.^11^ LC3 is a homolog of yeast Atg8 and is frequently used as autophagosome marker.^11^

Autophagy is involved in various physiologic processes, such as development, differentiation and aging.^14, 15^ Autophagy is essential to cardiac and tooth morphogenesis during vertebrate development.^16, 17^ A previous study reported that autophagy is a participant process in the osteogenic differentiation of mesenchymal stem cells (MSCs),^18^ M2 macrophage differentiation^19^ and muscle cell differentiation.^20^ Recent studies determined that autophagy is involved in the odontoblast aging process and that the longevity of odontoblasts is maintained by an elaborate autophagic–lysosomal system.^21, 22^ Autophagy modulation is also a potential therapeutic target for diverse illnesses, including cancer and metabolic, neurodegenerative, and infectious diseases.^23^ Numerous pathogens can be degraded by autophagy *in vitro*, and autophagy genes have a protective role against pathogens *in vivo*.^24, 25^

Our previous study showed that lipopolysaccharide (LPS) can induce autophagy in pre-odontoblasts and that autophagy is important to the survival of LPS-stimulated pre-odontoblasts.^26^ Our pilot study also suggested that LPS stimulation can enhance odontoblast differentiation capacity. LPS is a cell wall component of Gram-negative bacteria, which can activate the transcription factor nuclear factor-*κ*B (NF-*κ*B). NF-*κ*B has an important role in the regulation of inflammatory responses.^7^ A current study suggested that the reduced expression of NF-*κ*B in dental pulp stem cells in the presence of inflammatory cytokines enhances odontoblastic differentiation and collagen matrix formation.^27^ A previous study also showed that NF-*κ*B inhibits the bone formation of osteoblast stems from initial inflammatory environment.^28^ Nonetheless, the relationship between autophagy and NF-*κ*B remains vague, as well as their involvement in odontoblast differentiation with LPS stimulation.

In this study, we investigate the role of autophagy in regulating odontoblast differentiation capacity with inflammation and the possible mechanism. Autophagy is essential to the control of odontoblast differentiation in inflammatory environments; LPS induces p-NF-*κ*B activation, which in turn inhibits this differentiation. In addition, the nucleus translocation of this transcription factor was suppressed by autophagy. Thus, our findings present a novel pathway of regulating odontoblast differentiation by autophagy through the suppression of p-NF-*κ*B activation in inflammatory environments.

## Results

### Odontoblast differentiation is upregulated with LPS stimulation

**Figure 1:**
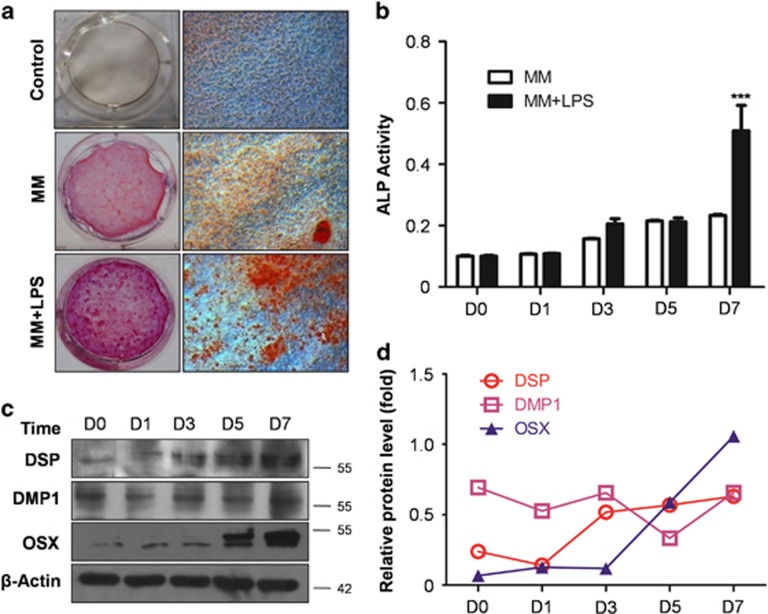
LPS enhances odontoblast differentiation capability. (**a**) mDPC6T were cultured in completed medium (Ctrl), MM and MM added LPS (MM+LPS) for 14 days. Alizarin red S was used to stain mineralization with red in the dishes. There are few red mineralized nodules in control group, whereas scattered mineralized nodules can be seen in MM. More minerals were detected in MM+LPS compared with MM group. (**b**) ALP activity in MM and MM+LPS group at different time points. It showed no significant difference between two groups within 5 days, nevertheless ALP activity was upregulated obviously in MM+LPS group at 7 days. Mean±S.E.M.; ***P<0.001. (**c**) Odontoblasts differentiation markers were detected with DSP, DMP1 and OSX during the process of differentiation in MM+LPS. The protein level of DSP, DMP1 and OSX were increased time dependently within 7 days. (**d**) Protein levels of DSP, DMP1 and OSX in the MM+LPS group were quantitated using densitometry. The experiments were repeated in triplicate, we analyzed every result of the experiments and made sure they showed the same pattern. Each target protein was normalized to the *β*-actin levels to provide a visible tendency, so the curve showed a relative protein level. Experiments were repeated at least twice

To determine odontoblast differentiation capability, mineralized nodules were stained with Alizarin red S after cells were cultured for 14 days. Red mineralized nodules were not observed in the control group, whereas scattered nodules were detected in mineralized-induced medium (MM). The amount of minerals detected in MM increased obviously with the addition of LPS (MM+LPS), as shown in Figure 1a. Meanwhile, alkaline phosphatase (ALP) activity was detected in MM with or without LPS treatment. No significant difference was observed between the two groups over a period of 5 days. Furthermore, ALP activity was obviously upregulated in the MM+LPS group on day 7 (*P*<0.001; Figure 1b). The expression levels of DSP, DMP1 and OSX were also upregulated during odontoblast differentiation with the incorporation of MM+LPS (Figures 1c and d). These results suggest that LPS can enhance odontoblast differentiation capacity in MM.

### Autophagy was increased in cells cultured with induced medium containing LPS

**Figure 2:**
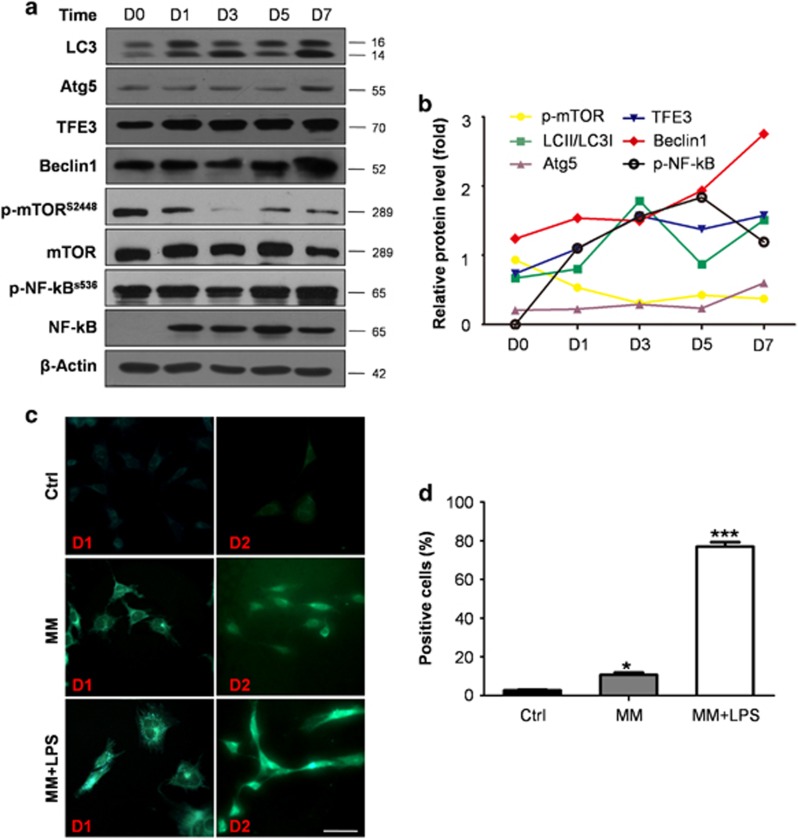
Autophagy was increased in the process of differentiation in inflammatory environment. (**a**) Autophagy-related marks, such as LC3, Atg5, TFE3 and Beclin1, were upregulated with time. The expression of p-mTOR showed an opposite trend with LC3, it decreased with time especially at 3 days, at which the ratio of LC3II to LC3I begin to upregulate obviously. P-NF-*κ*B increased immediately at d1, but the expression was negatively related with the expression of autophagy in later time. It decreased at 7 days, while autophagy peaked at 7 days. (**b**) Quantification of autophagy protein levels of the MM+LPS group using densitometry. LC3, Atg5, TFE3, Beclin1 were upregulated, p-mTOR was downregulated. P-NF-kB increased immediately with LPS treatment, but it decreased at 7 days. (**c**) mDPC6T were cultured in complete medium (Ctrl), MM, MM added LPS (MM+LPS) for 1 and 2 days, and the autophagosome can be detected with MDH. A few stained vacuoles were detected in cells cultured in MM, and the green-labeled vacuoles increased obviously in cells cultured in MM+LPS. Scale bar=50 *μ*m. (**d**) A total of 200 cells were calculated in each group and the positive cells were calculated in each group at 1 day. There is significant difference when comparing MM and LPS+MM with control group. The experiments were repeated in triplicate, we analyzed every result of the experiments and made sure they showed the same pattern. Each target protein was normalized to the *β*-actin levels to provide a visible tendency, so the curve showed a relative protein level. Mean±S.E.M.; *P<0.05; ***P<0.001

**Figure 3:**
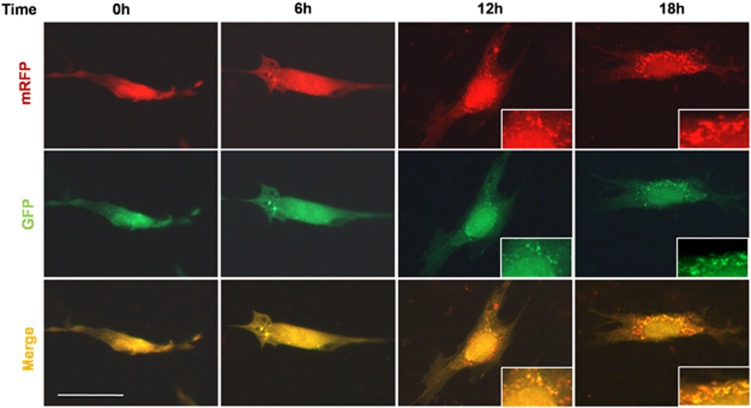
Autophagy flux was detected by mRFP-GFP-LC3 plasmid. Cells with pre-transfection of mRFP-GFP-LC3 were cultured with MM+LPS for 6, 12 and 18 h, and the puncta of LC3 was detected with fluorescence microscopy. The distribution of mRFP-GFP-LC3 was largely diffused, then showed puncta structures from 6 h and accumulated in cells time dependently. Both red and yellow puncta were increased in cells. Scale bar=50 *μ*m. Experiments were repeated at least twice

To further determine the possible reason for the upregulation of odontoblast differentiation capacity with LPS stimulation, cells were collected at different time points and the markers of differentiation and autophagy were detected. LC3 was upregulated in a time-dependent manner and increased considerably from day 3 onward. Beclin1 exhibited expression patterns that were similar to that of LC3; that is, it increased with time. Atg5 and TFE3 showed slight upregulation and peaked at day 7. Although the expression of p-mTOR was downregulated time dependently (Figures 2a and b). Phosphorylated NF-*κ*B expression increased immediately on day 1 but decreased on day 7 when the cells were cultured in MM plus LPS group (MM+LPS). This finding indicated that this transcription factor is negatively related to autophagy markers (Figures 2a and b). Few autophagosomes were detected in the complete medium, whereas stained vacuoles were found with monodansylpentane (MDH) in cells cultured in MM (Figure 2c). More green-labeled vacuoles were observed in cells cultured in MM+LPS than that in MM (Figures 2c and d). Cells with pre-transfected mRFP-GFP-LC3 were cultured in MM+LPS for 0, 6, 12 and 18 h. Autophagic flux was morphologically traced with an mRFP-GFP-LC3 tandem construct, and autophagosomes and autolysosomes were labeled in yellow and red, respectively. GFP fluorescence can be quenched given the low pH inside the lysosomes, whereas mRFP fluorescence is stable.^29, 30^ Yellow (autophagosomes) and red (autolysosomes) puncta were detected from 6 h onward and increased with time in the cells (Figure 3). This outcome indicated autophagy flux increased and that autophagosomes accumulated because of autophagy induction.

### Autophagy regulates odontoblast differentiation in inflammatory environment

**Figure 4:**
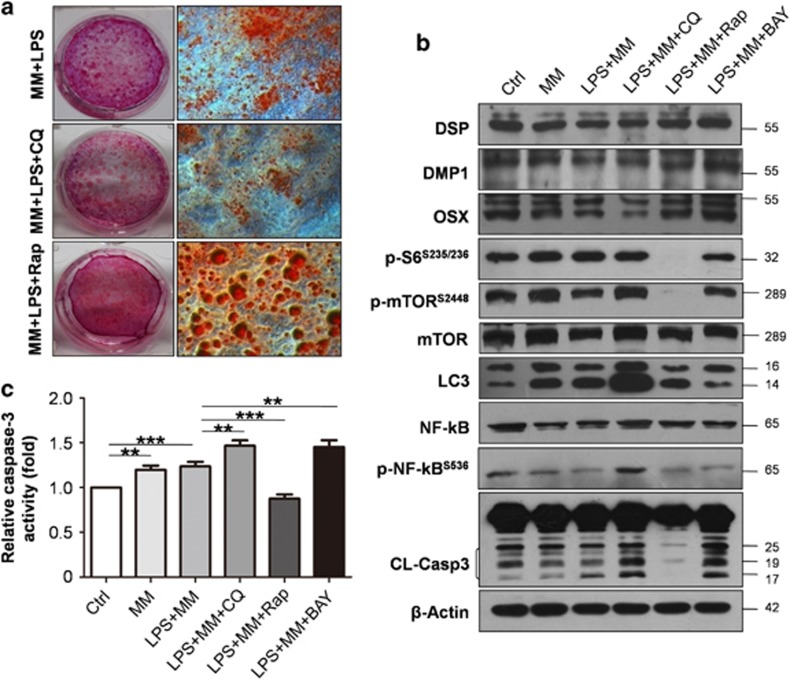
Autophagy regulates odontoblast differentiation in inflammatory environment. To confirm the role of autophagy in the upregulation of odontoblast differentiation with LPS treatment, CQ or rapamycin were used to block or enhance autophagy, respectively. (**a**) Mineralized nodules were decreased with autophagy inhibitor CQ, whereas the number and volume of nodules increased with rapamycin treatment. (**b**) Representative western blot shows accumulation of LC3II was found when using CQ to block the fusion of autphagosome and lysosome. The ratio of LC3II to LC3I was upregulated in MM+LPS+Rap group, whereas it decreased in MM+LPS+BAY group. When rapamycin was used, the expression of p-mTOR and p-S6 was decreased sharply. The protein levels of DSP, DMP1 and OSX were downregulated with CQ treatment, whereas their expression levels increased with rapamycin and BAY11-7082 treatment. Protein level of p-NF-*κ*B obviously increased when blocking autophagy with CQ, whereas it decreased in rapamycin (autophagy enhancer) and BAY11-7082 (p-NF-*κ*B inhibitor) treated group. It also showed increased cleaved caspase-3 (CL-Casp3) in CQ and BAY11-7082 treated group. *β*-Actin was used as a loading control. (**c**) Caspase-3 activity assay was detected in different medium. Caspase-3 was activated in MM and MM+LPS, and it was higher in CQ and BAY11-7082 treated group than that in MM+LPS group. It sharply decreased in MM+LPS+Rap group. Mean±S.E.M.; **P<0.01; ***P<0.001. Experiments were repeated at least twice

To confirm the role of autophagy in the upregulation capacity of differentiation in LPS-treated cells, autophagy was either blocked or enhanced with inhibitor chloroquine (CQ) or enhancer rapamycin, respectively. The number of mineralized nodules decreased when autophagy was inhibited by CQ, whereas the number and volume of nodules increased with rapamycin treatment (Figure 4a). The expression levels of DSP, DMP1 and OSX were downregulated with CQ treatment but were upregulated when autophagy was enhanced. LC3II accumulated with CQ treatment. Rapamycin induces autophagy in a variety of cell types by inhibiting the mammalian target of mTOR. The ratio of LC3II to LC3I increased, whereas expression levels of the mTOR signal-related proteins p-mTOR and p-S6 were downregulated with the addition of rapamycin (Figure 4b). The protein levels of DSP, DMP1 and OSX were upregulated when BAY11-7082 was used to inhibit NF-*κ*B activation. The protein level of cleaved caspase-3 also increased when treated with CQ and BAY11-7082 in MM+LPS; however, this level decreased under rapamycin treatment. Moreover, p-NF-*κ*B expression increased when autophagy was inhibited but decreased with rapamycin or BAY11-7082 treatment (Figure 4b). These results suggest that odontoblast differentiation capacity was elevated when autophagy was enhanced or NF-*κ*B activation inhibited. Meanwhile, autophagy and p-NF-*κ*B are negatively related. In addition, cleaved caspase-3 was activated when cells were treated with MM and MM+LPS as indicated by western blot (Figure 4b). The expression of cleaved caspase-3 was even higher with CQ and BAY11-7082 in MM+LPS compare with MM+LPS; however, it decreased sharply with rapamycin treatment (Figure 4b). Moreover, caspase-3 activity assay exhibit the same changes as the cleavage using Ac-DEVD-pNA as a caspase-3-like proteinase substrate (Figure 4c).

### Autophagy suppresses NF-*κ*B activation during odontoblast differentiation

**Figure 5:**
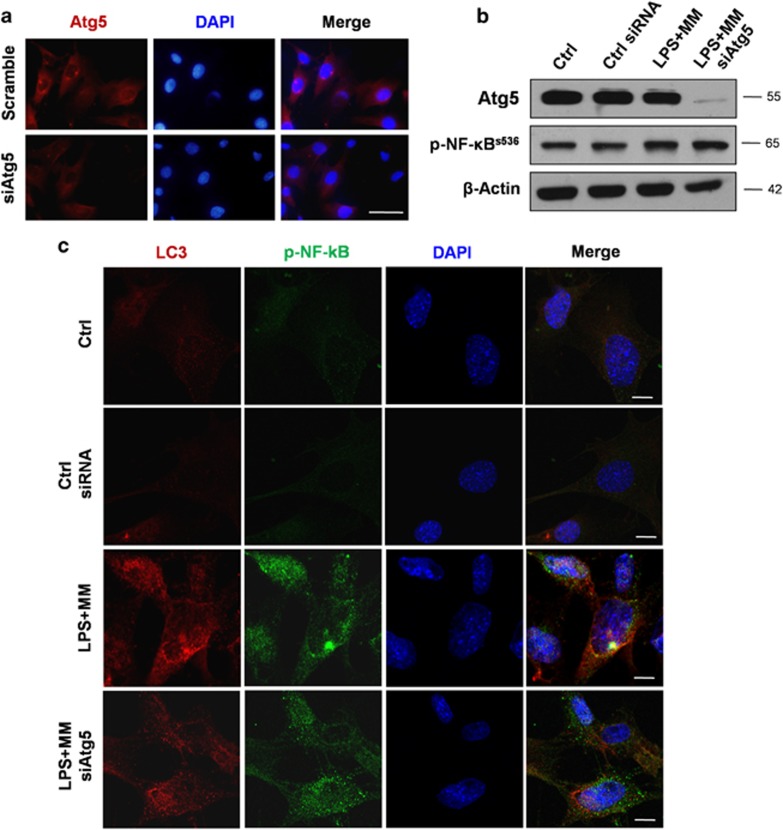
Autophagy can suppress the activation of NF-*κ*B during odontoblast differentiation. Cells were transiently transfected with Atg5 siRNA to inhibit autophagy. (**a**) The expression of Atg5 was decreased, after cells were transfected with Atg5 siRNA. Scale bar=50 *μ*m. (**b**) Cells transfected with control and Atg5 siRNA were cultured in MM+LPS for 3 days, then collected and lysed for western blot. Atg5 was knockdown in transfected cells, which was proved by western blot analysis. The protein level of p-NF-*κ*B was upregulated, when autophagy was inhibited. (**c**) Cells transfected with control or Atg5 siRNA were cultured with MM+LPS, and cells in complete medium were used as control group. Cells were fixed after culture for 1 day, and stained with anti- p-NF-*κ*B and LC3. The cellular distribution of these proteins was analyzed with confocal microscopy. LC3 was colocalized with p-NF-*κ*B in cells treated with MM+LPS. When autophagy was inhibited by siAtg5, the expression of LC3 was decreased, and the nucleus translocation of p-NF-*κ*B increased. Few expression of LC3 and p-NF-kB were detected in control and control siRNA group. Scale bar=10 *μ*m. Experiments were repeated at least twice

To investigate the relationship between autophagy and p-NF-*κ*B further, Atg5 small interfering RNA (siRNA) was transiently transfected to cells to inhibit autophagy. Atg5 immunofluorescence was detected in cells cultured in MM+LPS and weakened with siAtg5 transfection (Figure 5a). The protein level of p-NF-*κ*B increased during the Atg5 knockdown of cells, as per a western blot analysis (Figure 5b). Double immunofluorescence results showed that LC3 (red) and p-NF-*κ*B (green) were mainly colocalized in the cytoplasm of MM+LPS-cultured cells. Nonetheless, LC3 and p-NF-*κ*B were not clearly expressed in cells that were cultured in a complete medium (Figure 5c). LC3 expression decreased after siAtg5 transfection, whereas the nucleus translocation of p-NF-*κ*B increased (Figure 5c). These findings suggested that p-NF-*κ*B degradation is dependent on autophagy, which can suppress NF-*κ*B activation by preventing nucleus translocation.

### LC3 and p-NF-*κ*B were colocalized in tooth samples with deep caries

**Figure 6:**
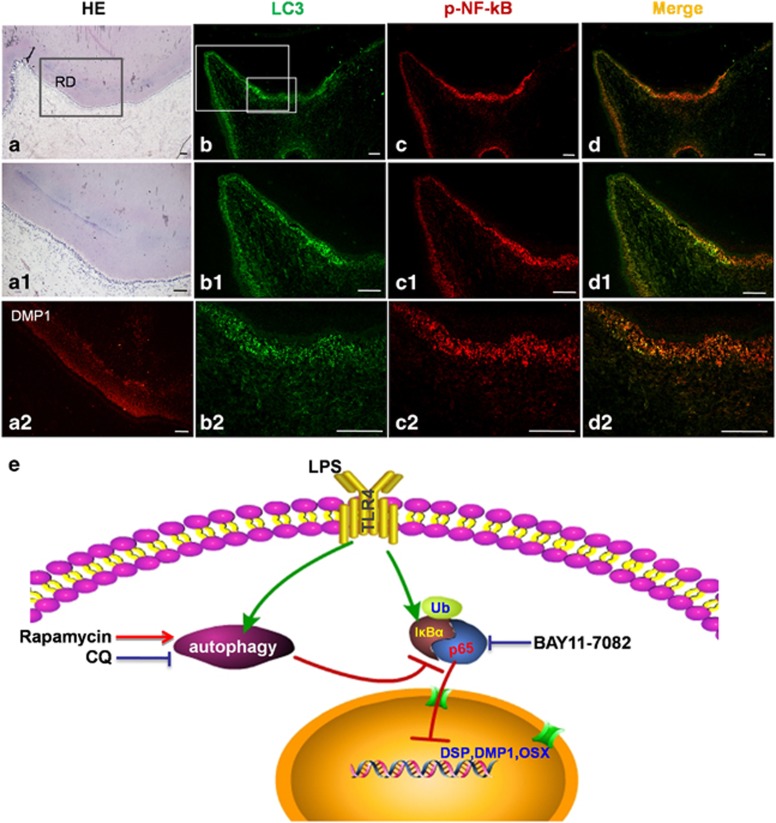
The colocalization of LC3 and p-NF-*κ*B in deep caries teeth with reactionary dentin. The images of immunofluorescence staining of p-NF-*κ*B (red) and LC3 (green) was analyzed by multiplexed molecular imaging. (**a-a2**) The tissue of reactionary dentin can be seen in caries tooth with HE stain, and the dentin of the teeth was labeled by DMP1 immunofluorescence. Scale bar=100 *μ*m. (**b-b2**) LC3 was expressed in the layer of odontoblasts, especially the cells at roof of pulp chamber. (**c-c2**) p-NF-*κ*B was detected in odontoblasts facing roof of pulp chamber in deep caries tooth. (**d-d2**) The colocalization of p-NF-*κ*B and LC3 can be found at the layer of odontoblasts, sub-odontoblastic layer and dental pulp cells near roof of pulp chamber. Scale bar=100 *μ*m. (**e**) Schematic of autophagy regulates odontoblast differentiation through suppression of NF-*κ*B activation. Autophagy and NF-*κ*B can be activated in cells with MM added LPS. The activation of NF-*κ*B can inhibit the capacity of odontoblast differentiation in inflammatory microenvironment. Autophagy degrades the p-NF-*κ*B, decreasing the nucleus translocation of p-NF-*κ*B and enhancing odontoblast differentiation capacity. CQ, blocking autophagy, inhibits odontoblast differentiation. Rapamycin, autophagy enhancer, upregulates odontoblast differentiation by suppressing p-NF-*κ*B nucleus translocation

Teeth with deep caries were obtained to verify LC3 and p-NF-*κ*B colocalization in human samples. Reactionary dentin was detected in these teeth via HE staining (Figures 6a–a1) and DMP1 immunofluorescence (Figure 6a2). P-NF-*κ*B, labeled with a QD-525 probe, was observed in odontoblasts that faced the roof of the pulp chamber; this area contained significant amounts of reactionary dentin (Figures 6c–c2). LC3 was extensively expressed in the odontoblast layer, in the sub-odontoblast layer, and in a few dental pulp cells, especially inflamed cells (Figures 6b–b2). LC3 and p-NF-*κ*B were colocalized in the odontoblast layer and in the dental pulp cells, thus confirming the colocalization process in the examined tooth samples (Figures 6d–d2).

## Discussion

Odontoblasts are terminally differentiated cells that are subject to a long-lived secretory condition and mediate dentinogenic activity.^31^ Reactionary dentin is formed as an adaptive secretory response when the occurrence of moderate dentin injury stimulates odontoblast generation.^31, 32^ In this study, we investigate odontoblast differentiation with LPS stimulation and its mechanism (Figure 6e). LPS stimulates odontoblast differentiation capacity, and the mechanism of this phenomenon is related to enhanced autophagy. Moreover, inflammation induced the upregulation of autophagy, which promoted odontoblast differentiation capacity by suppressing NF-*κ*B nuclear translocation and activation.

Odontoblast differentiation capacity was upregulated by medium-dose exogenous LPS stimulation. This result was confirmed by Alizarin red staining, ALP activity analysis results and the increased expression of differentiation markers. A previous study reported that tumor necrosis factor-*α* (TNF-*α*) can stimulate the differentiation of dental pulp cells to odontoblastic phenotypes through p38.^33^ Goldberg *et al.*^32^ pointed out that osteoblast/odontoblast-like differentiation can be promoted by activating dendritic cells via a mild inflammatory process. These studies suggest that the injury may influence odontoblast differentiation to overexpress dentinogenic markers and enhance secretory behavior, which are consistent with our results in mDPC6T and caries teeth. Other studies determined that inflammatory microenvironments limited the osteogenic differentiation of periodontal ligament stem cells (PDLSCs).^34^ MSC osteoblast differentiation capacity decreased in patients or animals with rheumatoid arthritis,^35^ whereas it was enhanced with IL-17 stimulation.^36^ Therefore, the effect of inflammation on differentiation capacity may be cell specific. Nonetheless, the mechanism of odontoblasts differentiation and reactionary dentin production in inflammatory microenvironments remains vague.

Autophagy is involved in various cell differentiation procedures, such as osteogenic differentiation of MSC, macrophage and muscle cell differentiations.^18, 19, 20^ In our study, autophagy was found regulated in odontoblast differentiation with LPS stimulation. This process improved with the inclusion of upregulated autophagy-related markers, autophagosomes and autophagy flux. MDH staining showed that autophagy was observed during odontoblast differentiation, and even more in cells with LPS stimulation. These results suggested that autophagy aids in the regulation of odontoblast differentiation with LPS stimulation. Odontoblast differentiation capacity decreased with autophagy inhibition but increased with rapamycin treatment. A previous study implied that rapamycin can stimulate the osteoblastic differentiation of hMSCs by modulating mTOR and BMP/Smad signaling.^37^ Moreover, autophagy suppression through FIP200 deletion resulted in the inhibition of formation and differentiation of osteoblast nodules.^38^ Autophagy is essential to odontoblast differentiation in inflammatory environments given that differentiation capacity is enhanced by autophagy activation. This outcome suggests that autophagy can regulate odontoblast differentiation in defense against bacterial invasion, and sub-odontoblast layer cell differentiated into odontoblast-like cell and produce reactionary dentin.

We also further explored the mechanism of autophagy-regulated odontoblast differentiation and determined that autophagy regulates odontoblast differentiation by suppressing NF-*κ*B activation. During odontoblast differentiation in MM+LPS, NF-*κ*B activation was negatively related with autophagy. It was also found that NF-*κ*B activation may inhibit the odontoblast differentiation in inflammatory microenvironment. Previous studies reported that IKK/NF-*κ*B inhibition can help promote bone formation in the treatment of osteoporosis and bone diseases.^39^ NF-*κ*B is central to the regulation of PDLSC osteogenic differentiation in inflammatory microenvironments.^40^ LPS can trigger NF-*κ*B activation and facilitate p65/p50 translocation into nucleus, where it activates the transcription of NF-*κ*B.^41^ The nuclear translocation of NF-*κ*B was detected in cells cultured with MM+LPS, whereas p-NF-*κ*B and LC3 were colocalized in the cytoplasm. NF-*κ*B translocation increased when autophagy was inhibited by Atg5 siRNA. These results verified that autophagy can suppress the nuclear translocation of NF-*κ*B, which inhibits odontoblast differentiation capacity. Furthermore, LC3 was clearly detected in the infected odontoblast, as was the colocalization of p-NF-*κ*B and LC3 in the odontoblasts and sub-odontoblastic layer of caries teeth with reactionary dentin. Previous study showed that a reduction of autophagic activity in aging odontoblasts with lipofuscin accumulation.^22^ In our study, autophagy was activated in young odontoblast in response to cariogenic bacteria infection. The functional viability of odontoblasts is activated with autophagic activity in young tooth. These findings provide new insight into the mechanism that facilitates the significant role of autophagy in inducing infected odontoblasts to produce reactionary dentin in defense against invasion.

The host experiences different modes of cell death during infection, including apoptosis, pyroptosis and necrotosis mediated by caspases.^42^ Caspase-3, -6 and -7 mediates cell death as an apoptotic effector/executioner. A recent study showed that NF-*κ*B is responsible for inflammatory mediators and survival proteins; moreover, certain pathogens modulate apoptosis by interfering with NF-*κ*B expression.^43^ In this study, cleaved caspase-3 was activated when cells were treated with CQ and BAY11-7082 in MM+LPS; however, cleaved caspase-3 expression decreased sharply with rapamycin treatment. Differentiated odontoblasts almost died on day 14 when treated with BAY11-7082 (data not shown), thereby suggesting the importance of autophagy and NF-*κ*B in the survival mechanism of differentiated odontoblast in inflammatory environments. The expression levels of numerous pro-inflammatory cytokines increased during inflammatory response, including those of TNF-*α* and interleukin-1.^44^ A current study reported that necroptosis can suppress inflammation by terminating the production of either TNF or an LPS-induced cytokine and chemokine.^45^ Nonetheless, the process of cell death during odontoblast differentiation with the invasion of inflammation warrants further exploration.

In conclusion, our study suggested autophagy-regulated odontoblasts differentiation capacity in inflammatory environment. Autophagy could suppress the activation of NF-*κ*B, which inhibited odontoblasts differentiation. We first put forward that autophagy regulates odontoblast differentiation to defense the inflammation. These results provided new insight into the defense mechanisms of the dentin–pulp complex during inflammation invasion, and this knowledge may be useful for the therapy of caries and pulpitis and dental pulp regeneration in inflammatory environments.

## Materials and Methods

### Cell culture and differentiation

mDPC6T is a self-established cell line derived from mouse dental papilla,^4, 46^ which maintains similar phenotype and function of primary mouse dental papilla cells. They exhibit spindle shape, express identification markers of odontoblastic-related genes and have mineralization ability. mDPC6T was grown in *α*-modified Eagle's medium (*α*-MEM, Gibco-BRL Life Technologies, Paisley, UK) containing 10 % fetal bovine serum (Invitrogen, Carlsbad, CA, USA) and cultured in air containing 5% CO_2_ at 37 °C.

For odontoblastic induction, cells were cultured as described previously.^4, 47^ Briefly, mDPC6T were seeded in six-well plates (Falcon, Franklin Lakes, NJ, USA) with a density of 2 × 10^5^ cells per well. The cells were cultured in MM, which is *α*-MEM containing 10% FBS, 50mg/ml ascorbic acid, 10 mM sodium *β*-glycerophosphate and 10 nM dexamethasone (Sigma, St Louis, MO, USA). Protein were extracted for western blot analysis and ALP activity assays (Supplementary Information).

### Western blot

Western blot were performed as previously described (Supplementary Information).^26^ Quantification of protein levels was performed with Image J software (National Institutes of Health, Bethesda, MD, USA). Each target protein was normalized to the *β*-actin levels, so the curve showed a relative protein level.

### Alizarin red staining

After mDPC6Ts were cultured in odontoblastic induction medium for 14 days, mineralized nodules were detected by staining with Alizarin red (Sigma). Briefly, the cells in six-well plates were washed with phosphate-buffered saline (PBS) and fixed with 95% ethanol for 10 min. Cells were stained with 1% Alizarin red dissolved in distilled water, pH 5.5 at room temperature with gentle agitation for 30 min. Cells were washed with distilled water to remove the unbound Alizarin red. The stained cells were photographed with an inverted phase-contrast microscope (OLYMPUS IX41, Olympus, Shinjuku-ku, Tokyo, Japan).

### ALP activity assay

mDPC6T was cultured in MM with or without LPS for 0, 1, 3, 5 and 7 days, and cells were collected with lysis buffer for ALP *in vitro* chemical assays. ALP activity was detected in the cell lysate using ALP substrate buffer containing p-nitrophenyl phosphate (Sigma) for 50 min at 37 °C. The reaction was stopped by 10 mM NaOH, and the absorbance was read at 405 nm. ALP activity was calculated as 1 mol of p-nitrophenol per mg protein.

### MDH labeling of autophagosome

Cells were seeded on coverslips and cultured in complete medium (Ctrl), MM, MM added LPS (MM+LPS) for 1 and 2 days, and then autophagic vacuoles were stained with MDH. Immediately analysis was taken by fluorescence microscopy using an inverted microscope equipped with a filter system (Supplementary Information).

### Transient transfection of mRFP-GFP-LC3 plasmid

Cells grown on glass coverslips were fixed in 2% formaldehyde for 10 min at room temperature. Detection of autophagosomal structures was performed by fluorescence microscopy observing LC3B puncta in cells. Autophagic flux was analyzed by fluorescence microscopy monitoring the distribution and alteration of mRFP-GFP-LC3B fluorescent signals.

### Caspase-3 activity assay

Cells were seeded in six-well plates, and cultured with MM, LPS+MM, LPS+MM+CQ, LPS+MM+rapamycin and LPS+MM+BAY11-7082 for 3 days. Complete medium was regarded as control group. Caspase-3 activity was detected with caspase-3 activity assay kit (Beyotime, Jiangsu, China) according to the manufacturer's instructions. Briefly, cell lysates were mixed in 100 *μ*l reaction buffer containing 10 *μ*l caspase-3 substrate (Ac-DEVD-pNA) (2 mM) and incubated at 37 °C. The concentration of pNA was measured with a reader at an absorbance of 405 nm. Results were expressed as relative caspase-3 activity (fold).

### Transfection and cell immunofluorescent double staining

Small inhibitory RNA against Atg5 (siAtg5, Sigma) was injected to inhibit autophagosome formation. Atg5 RNA interference was accomplished by transfecting mDPC6T with Atg5-targeted siRNA and the Universal Control siRNA. The cells were grown in six-well plates and then transfected with FuGENE HD (Roche Diagnostics, Mannheim, Germany) according to the manufacturer's instructions. Cells were collected and cell lysates were subjected to immunoblotting. Cells were cultured on coverslips in MM with or without LPS for 1 day, after transfecting with siAtg5. For immunofluorescence staining, cells transfected with Atg5 or control siRNA were fixed with 4% paraformaldehyde and incubated with anti-LC3 (1:100, Sigma) and p-NF-*κ*B p65 (1:100, Cell Signaling Technology, Danvers, MA, USA) antibodies, followed by secondary antibody conjugated with Fluor Cy3 or 488. The distribution of proteins in cell was observed with confocal fluorescence microscope (Olympus FV 1000).

### Samples and QDs-based multiplexed molecular imaging

Human third molars with morbidity, such as pain, infection and swelling, were removed from patients between 20 and 25 years from the School and Hospital of Stomatology, Wuhan University. The procedures were performed based on the guidelines of the National Institutes of Health regarding the use of human tissues and with permission from the Institutional Ethical Board of Wuhan University. Teeth with caries were fixed in 4% buffered paraformaldehyde, decalcified in 10% ethylene diaminetetraacetic acid/PBS solution and then embedded in paraffin. The paraffin block-embedded tissue was cut into 4 *μ*m sections, deparaffinized, rehydrated and antigen retrieved with microwave. After being blocked with 2.5% bovine serum albumin (BSA) for 1 h at 37 °C, the slides were incubated with the primary antibody for p-NF-*κ*B (1 : 400, Cell Signaling Technology) overnight at 4 °C, and then incubated with secondary antibody conjugated with QD-525 (QDs-525 goat F(ab')2 anti-rabbit IgG conjugate, Invitrogen) for 1h. In all, 2.5% BSA was used to block the slides again after washing. Primary antibody LC3 (1 : 100, Sigma) was incubated for 2 h at 37 °C, then biotinylated IgG for 0.5 h, QD-605 conjugated streptavidin for 2 h. Multiplexed molecular imaging was used to analyze the results.

### Statistical analysis

All data were presented as the mean±S.E.M. Data were analyzed and visualized using Graph Pad Prism 5.0 (GraphPad Software, La Jolla, CA, USA). Two-way ANOVA followed by Dunnett post-test were used to analyze ALP activity in MM without or with LPS at 0, 1, 3, 5 and 7 days. All experiments were independently repeated were repeated in triplicate. *P*<0.05 was regarded as statistically significant.

## Abbreviations

LPS: lipopolysaccharide
CQ: chloroquine
NF-*κ*B: Nuclear factor kappa B
TNF-*α*: tumor necrosis factor *α*
MDH: monodansylpentane
MM: mineralized-induced medium
siRNA: small interfering RNA
DSP: dentin sialoprotein
DMP1: dentin matrix protein-1
OSX: osterix
