# Supplementary material for: Autophagy regulates odontoblast differentiation by suppressing NF-κB activation in an inflammatory environment
Source: Cell Death Dis. 2016 Mar 3;7(3):e2122–. doi: 10.1038/cddis.2015.397 (PMC4823923; doi:10.1038/cddis.2015.397)
Supplement: Supplementary Materials [file cddis2015397x1.docx]

**Supplementary information:**

**Materials and Methods**

**Cell culture and differentiation** mDPC6T is a self-established pre-odontoblast cell line, which maintains similar phenotype and function of primary mouse dental papilla cells. They exhibit spindle shape, express identification markers of odontoblastic-related genes and have mineralization ability. mDPC6T was grown in α-Modified Eagle's Medium (α-MEM, Gibco-BRL Life Technologies, Paisley, UK) containing 10 % fetal bovine serum (Invitrogen), and cultured in air containing 5% CO2 at 37˚C.

For odontoblastic induction，cells were cultured as described previously. Briefly, mDPC6T were seeded in 6-well plates (Falcon, Franklin Lakes, NJ , USA) with a density of 2×10^5^ cells per well. The cells were cultured in mineralized induced medium, which is α-MEM containing 10% FBS, 50mg/ml ascorbic acid, 10mM sodium β-glycerophosphate, and 10nM dexamethasone (Sigma, MO, USA). The cells cultured in α-MEM with 10% bovine serum were used as a blank control. Meanwhile, the cells grown in mineralized induced medium were treated with 1 µg/mL LPS (Sigma, MO, USA), 10 μM autophagy inhibitor, CQ (Sigma, MO, USA), 100nM autophagy enhancer, Rapamycin (Sigma, MO, USA), 15μM NF-κB inhibitor, BAY11-7082. Protein were extracted for Western blot analysis and alkaline phosphatase (ALP) activity assays.

**Western blot** Western blot were performed as previously described. Total proteins were extracted with lysis buffer containing protease inhibitor cocktail from Roche Diagnostics. Protein concentration was determined using the BCA Protein Assay Kit (Pierce Biotechnology, Rockford, IL). Protein extracts were separated on 8% to 12% SDS-polyacrylamide gel electrophoresis, and then transferred to polyvinylidene ﬂuoride membrane (Millipore). After the membranes were blocked with 5% nonfat milk for 1h at room temperature, primary antibodies were used at 1/1000-1/2000 dilution: anti-Beclin1 (1:2000, Novus), anti-Atg5 (1:1000, Cell signaling), anti-cleaved Caspase3 (1:1000, Cell signaling), anti-LC3 (1:2000, Sigma), anti-p-mTOR^S2448^ (1:1000, Cell signaling), anti-p-S6^S235/236^ (1:1000, Cell signaling), anti-NF-κB (1:1000, Cell signaling), anti-p-NF-κB ^s536^ (1:1000, Cell signaling), anti-TFE3 (1:1000, Novus), anti-DMP1 (1:2000, Santa Cruz), anti-DSP (1:1000, Santa Cruz), anti-OSX (1:1000, Santa Cruz). Then, the membranes was incubated with horseradish peroxidase-conjugated secondary antibody (Santa Cruz Biotechnology, CA, USA) for 1 h. Quantiﬁcation of protein levels was performed with Image J software. Each target protein was normalized to the β-actin levels, so the curve showed a relative protein level.

**MDH labeling of autophagosome** Cells were seeded on coverslips and culture in complete medium (Ctrl), mineralized induced medium (MM), mineralized induced medium added LPS (MM+LPS) for 1d, 2d, and then autophagic vacuoles were stained with monodansylpentane (MDH). Briefly, cells on glass coverslips were washed with PBS, and incubated at 37℃ for 30min with 200μM MDH in darkness. Cells were fixed with 4% paraformaldehyde for 15 min, and washed with PBS. Immediately analysis was taken by fluorescence microscopy using an inverted microscope equipped with a filter system.
